# Effects of Oxytocin Administration on the Response of Piglets to Weaning

**DOI:** 10.3390/ani5030371

**Published:** 2015-07-16

**Authors:** Jean-Loup Rault, Frank R. Dunshea, John R. Pluske

**Affiliations:** 1 Faculty of Veterinary and Agricultural Sciences, University of Melbourne, Parkville, VIC 3010, Australia; E-Mail: fdunshea@unimelb.edu.au; 2 School of Veterinary and Life Sciences, Murdoch University, Murdoch, WA 6150, Australia; E-Mail: j.pluske@murdoch.edu.au

**Keywords:** behavior, intranasal, oxytocin, physiology, stress, subcutaneous, sus scrofa

## Abstract

**Simple Summary:**

Weaning is a stressful milestone for domestic animals. It is often performed at an early age and as an abrupt change in comparison to the transitional period seen in feral or wild animals. Oxytocin, a hormone associated with attachment, could improve the response of piglets to weaning. Piglets were either given oxytocin intranasally, subcutaneously, or handled as controls. Oxytocin had no effect on the physiological response to weaning. However, oxytocin increased the frequency of mild aggressive social behaviors between OT-administered and control pigs. Hence, the use of a single administration of oxytocin prior to weaning in pigs is not recommended.

**Abstract:**

Weaning is often an abrupt and stressful process. We studied the effects of administering oxytocin, subcutaneously or intranasally, on the ability of pigs to cope with weaning. On a commercial farm 144, 30 day-old pigs from 24 litters were used. On the day of weaning, one male and one female in each litter were administered one of three treatments: intranasal oxytocin (24 International Unit), subcutaneous oxytocin (10 International Unit per kg of body weight), or handled as a control. The pigs were placed in one of eight weaner pens, split by sex and with an equal representation of treatments. Data included body weight and growth, physiology (neutrophil:lymphocyte ratio, plasma cortisol, C-reactive protein and Tumor Necrosis Factor-α concentrations), and behavior (feeding, drinking, social behavior). Both oxytocin treatments tended to result in higher levels of mild aggression within groups (*p* = 0.08), specifically between oxytocin-administered and control pigs (subcutaneous to control *p* = 0.03; intranasal to control *p* = 0.10). Subcutaneously-administered pigs tended to frequent the feeder more often than intranasally-administered pigs (**p* <* 0.10), with the latter having slightly lower body weight 38 days post-weaning (*p* = 0.03). However, acute oxytocin administration did not result in any noticeable physiological changes 4 or 28 h post-weaning. Hence, the use of a single administration of oxytocin prior to weaning in pigs is not recommended, at least not in the conditions studied here.

## 1. Introduction

The practice of weaning domestic animals drastically differs from the transitional period seen in feral animals or their wild ancestors. In commercial pig production for instance, weaning is commonly performed around three to four weeks of age, whereas it takes between 8 and 19 weeks to complete in feral pigs [1]. In addition to the age difference, weaning most often consists in an abrupt process with a transition from liquid (e.g., dam’s milk) to solid foods, along with a change in environment. Hence, weaning results in nutritional, thermal, immunological, and psychological challenges [2,3]. This commonly leads to weight loss and increased morbidity and mortality reflecting the young’s difficulty in coping with weaning [4]. Various strategies have been employed in order to make the weaning transition smoother such as weaning at older ages [5], providing supplemental milk and creep feed during lactation [6,7], using metabolic modifiers or gut health-promoting diets [8,9], and socializing animals prior to weaning [10].

Oxytocin (OT) is an endogenous mammalian hormone which plays a crucial role in maternal behavior, allowing milk let down and parturition through its peripheral actions but also underlying attachment through its central actions [11,12,13]. We hypothesized that OT may facilitate the weaning process. In rats, OT administration to pups reduces time spent with the dam and the response to social isolation, while it increases self-oriented behaviors, post-weaning feed intake and growth [14,15,16,17]. Oxytocin has also been shown to be involved in the stress response of pigs [18]. A previous project [19] investigated the effects of repeated OT administration to neonatal pigs. Oxytocin subcutaneous administration daily for the first two weeks of life resulted in lower weight loss over the first two days post-weaning. The present study followed on that work by investigating the use of a single dose of OT administered immediately prior to weaning, and by comparing the effects of the intranasal and subcutaneous route of administration.

## 2. Experimental Section

### 2.1. Animals and Treatments

The project was approved by the University of Melbourne Animal Ethics Committee in accordance with the Australian Code of Practice for the Care and Use of Animals for Scientific Purposes. This study was conducted at a farrowing and weaner unit of a large commercial farm.

One hundred and forty four pigs from 24 litters, aged 30.6 ± 0.2 (mean ± SE) days were used. All pigs were the progeny of Large White × Landrace multiparous sows. Sows were housed in conventional 2.7 × 1.5 m farrowing crates, with the exception of three litters which were housed in 2.8 × 2.2 m farrowing crates (this had no influence on feeding frequency F_(1,119)_ = 1.59, *p* = 0.21 for instance between the two types of pens, and only one of those pigs was observed as an IN treatment for social behavior). Piglets were not provided with creep feed during lactation. In each litter, on the day prior to weaning (day 0), pigs were individually weighed and marked using plastic colored ear tags. Treatments were then allocated balancing for body weight across treatments. Males were not castrated as is common practice in other countries. The farrowing house was kept around 19 °C, with the creep area for piglets at 28 °C, and with light on from 0700 to 1600 h.

On the day of weaning (day 1), six pigs (three males and three females) were removed from their crate according to the normal weaning process around 0800 h. During the handling process, one male and one female from each litter were administered with either of three treatments: intranasal OT (‘IN’), subcutaneous OT (‘SC’), or simply handled as a control. The first treatment consisted of 24 International Unit of OT delivered intranasally (equivalent to 50 µg; peptide content 82%, peptide purity > 95% , Auspep, Tullamarine, VIC, Australia), diluted in 0.5 mL of 0.9% saline with 0.25 mL in each nostril, using a validated procedure [18]. The treatment was delivered using a Mucosal Atomizer Device (MAD 300, Wolfe Tory Medical Inc., Salt Lake City, UT, USA) connected to a 1 mL syringe, with the pig maintained in a head-up position. If the pig expelled the solution, a second administration (half-dose) was delivered in that nostril, which occurred in 6 out of 48 IN pigs. The second treatment consisted in 10 International Unit per kg of body weight of OT subcutaneously (equivalent to 21 µg per kg; Ilium Syntocin, Troy Laboratories, Glendenning, NSW, Australia), injected in the neck area behind the ear, with a half dose on each side, using a validated procedure [19]. Because pigs weighed about 8 kg, the SC dose was about three times higher than the IN dose of oxytocin. The third treatment consisted in a control treatment for which the pig was held for the same amount of time as for the two previous treatments with its nose touched. Treatments on average required handling the pigs between 30 and 45 sec, and pigs were individually marked with colored livestock spray paint according to their treatment.

The pigs were then separated per sex according to the farm’s practices, transported to another shed about 200 m away using a trolley, and placed into one of eight 3.4 m × 1.6 m weaner pens of 18 pigs per pen by mixing six litters together, hence with an equal representation of each treatment. Each pen contained a five-head space feeder and a nipple drinker located at each end of the pen. At the time of placement, pigs were administered a Mycoplasma hyopneumoniae vaccination given intramuscularly (RespiSure ONE, Pfizer Animal Health, West Ride, NSW, Australia). The weaner shed was kept around 26 °C, and with light on from 0700 to 1600 h. Pigs were finally transferred 31 days after weaning from the weaner to a grower facility located 150 km away.

### 2.2. Weight Measurements

Pigs were individually weighed one day prior to weaning, and 2, 7, 20 and 38 days after weaning using a portable scale with the weight recorded to the second digit.

### 2.3. Behavioral Observations

Behaviors were recorded through video cameras (GoPro model Hero3 white edition, GoPro Inc., San Mateo, CA, USA) placed above each of the weaner pens. Video recordings were analyzed for feeding, drinking and social behavior according to an ethogram (Table 1). Feeding and drinking behavioral observations were conducted by a single observer using a continuous recording method for 4 h after weaning and recording the frequency and duration of visits at the feeder and contacts with the nipple drinker with The Observer software (version 8.0, Noldus, the Netherlands). Social behavioral observations were conducted by a different observer using a continuous recording method for a 1 h period starting 30 min after weaning and recording the frequency of highly aggressive, mildly aggressive, or non-aggressive social behaviors performed by the focal pig, whether it was the initiator or recipient of that social exchange, and the treatment of the pig with which the focal pig interacted. Although the classification of social interactions as highly aggressive or mildly aggressive may seem arbitrary, it allowed to distinguish between brief negative social interactions (classified as mildly aggressive) and more pronounced or sustained negative social interactions (duration ≥ 5 sec or intensity ≥ two bites, classified as highly aggressive). For social behaviors, only half the pigs in each pen (n = 9 per pen), selected at random, were studied with three pigs from each treatment per pen and a total of 72 focal pigs. Social behaviors were recorded using Microsoft Excel. All observers were blind to treatments.

**Table 1:** Ethogram for behavioral observations, adapted from [18].

Behaviour	Description
**Maintenance behaviors**	
Feeding	Head in the feeder with both ears not visible
Drinking	Snout in physical contact with the drinker
**Social behaviors**	
High aggression	Bout lasts for ≥ 5 sec of head knock, pursuit, parallel push and/or ≥ two bites is delivered to the penmate
Mild aggression	Bout lasts for < 5 sec of head knock, pursuit, parallel push and/or one bite is delivered to the penmate
Non-aggressive	Snout to snout contact or any other touch with the snout of any penmate’s body parts

### 2.4. Physiological Analyses

Blood samples were collected from half of the pigs in each pen (n = 72) 4 h after weaning on day 1, selected at random, and from the other half of the pigs in each pen (n = 72) 28 h after weaning on day 2, to study the change of physiological parameters over time with blood collected at the same time of the day across both samplings and balanced across treatments. Blood samples were withdrawn via jugular venipuncture through manual arm restraint with a bleeder and a second person holding the pig, within 2 min which is not expected to influence basal plasma cortisol concentrations [20]. Blood samples were collected in 10mL lithium heparin tubes (BD vacutainer, North Ryde, NSW, Australia) that were then stored on ice. Blood samples for the peripheral leukocyte populations were kept at 4 °C and run through a blood analyzing system (Cell Dyn 3700, Abbott Diagnostics, Abbott Park, IL, USA) within 3 h of collection to determine the neutrophil:lymphocyte ratio. The rest of the blood samples were centrifuged with the plasma fraction transferred to microtubes for long-term storage at −20 °C. All samples were assayed in duplicate. Plasma cortisol concentrations were quantified using a commercial ELISA kit (Enzo Life Sciences, Farmingdale, NY, USA). Samples were diluted to 1:16 to fall within the standard curve and the sensitivity of the assay was 156 pg/mL. Mean intra- and inter-assay CVs were <6% and <9%, respectively. Plasma C-reactive protein (CRP) was measured using a commercial ELISA kit (Phase Porcine CRP Assay Kit, Tri Delta, Maynooth, Ireland). Samples were diluted to 1:800 to fall within the standard curve and the sensitivity of the assay was 46.9 ng/mL. Mean intra- and inter-assay CVs were <4% and <9%, respectively. Plasma Tumor Necrosis Factor α (TNF α) concentrations were quantified using a commercial ELISA kit (Porcine TNF α ELISA kit, Thermo Fisher Scientific Inc., Rockford, IL, USA). The sensitivity of the assay was 31.3 pg/mL. Only 62 samples were analyzed for TNF α (IN: n = 20; SC: n = 22; Controls: n = 20), and 30 of these samples returned values below the 31.3 pg/mL detection threshold (IN: n = 11; SC: n = 10; Controls: n = 9).

### 2.5. Statistical Analyses

All data were checked for normality and homogeneity of variance. Square root transformation was necessary for the duration of feeder visit. Data were subsequently analyzed using a mixed model (PROC MIXED) with the SAS statistical software (version 9.3, SAS Inst. Inc., Cary, NC, USA). The model included litter, treatment, sex, and their interaction if significant. The experimental design can be considered a 3 × 2 factorial design with the effects of treatment and sex. Weaning pen was considered the experimental unit for the effect of sex, and pigs nested within pens was considered the experimental unit for the effect of treatment, with the error terms chosen accordingly for each effect [21]. For the physiological variables, time bled after weaning (4 or 28 h) was added to the model, and its interactions with treatment and sex. For body weight and growth rate, measurements were accounted as repeated measures using day after weaning in the repeated statement in SAS. When significant interactions (*p* < 0.05) were detected, adjustments to the level of significance of the *p*-value were used to account for the number of pairwise comparisons between treatments on specific days or between days for specific treatments using Tukey-Kramer adjustments. Pearson correlations of SAS (PROC CORR) were conducted between physiological variables. Data are presented as least square means ± SE unless otherwise stated.

## 3. Results

### 3.1. Body Weight and Growth

Because treatments were allocated balancing for body weight, there were no differences in body weight prior to weaning (means ± SE: Intranasal OT: male 8.5 ± 1.5 kg, female 8.1 ± 1.4 kg; Subcutaneous OT: male 8.1 ± 1.5 kg, female 7.9 ± 1.3 kg; Controls: male 8.3 ± 1.5 kg, female 7.9 ± 1.3 kg).

**Figure 1:**
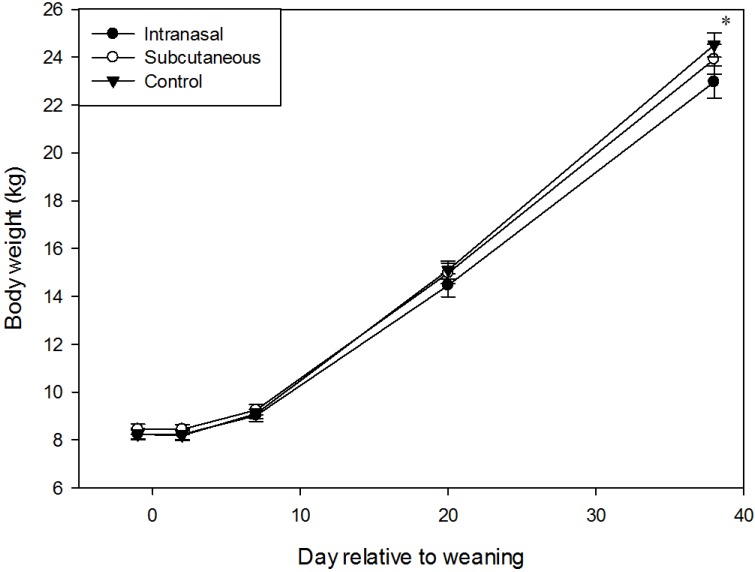
Body weight according to day relative to weaning and treatment (Least-square means ± standard errors; * *p* < 0.05). *N* = 48 per treatment.

Body weight did not differ according to treatment (F_(2,12)_ = 0.99, *p* = 0.40; Figure 1), or sex (F_(1,6)_ = 0.76, *p* = 0.42). Body weight differed according to the day (F_(4,72)_ = 1344.92, *p* < 0.001), and tended to differ according to the interaction of treatment and day (F_(8,72)_ = 1.75, *p* = 0.10), with IN pigs being lighter than control pigs 38 days after weaning (*p* = 0.01; Figure 1). Body weight also differed according to the interaction of sex and day (F_(4,72)_ = 2.35, *p* = 0.06), with females tending to be lighter than males 20 days after weaning (14.4 ± 0.3 kg *vs.* 15.2 ± 0.3 kg, respectively, *p* = 0.06).

Growth rate did not differ according to treatment (F_(2,12)_ = 1.63, *p* = 0.23), or sex (F_(1,6)_ = 0.03, *p* = 0.87), but differed according to the interval between sampling days (F_(3,60)_ = 486.31, *p* < 0.001), and the interaction of sex and interval (F_(3,60)_ = 6.43, *p* < 0.001). Females had a faster growth rate than males between 20 and 38 days after weaning (538.7 ± 15.5 g/d *vs.* 455.7 ± 15.5 g/d, respectively, *p* = 0.004).

### 3.2. Physiology

#### 3.2.1. Cortisol

Plasma cortisol concentration did not differ according to treatment (F_(2,12)_ = 0.34, *p* = 0.72; Table 2), or sex (F_(1,6)_ = 0.46, *p* = 0.53). Plasma cortisol concentration differed according to the time after weaning (F_(1,15)_ = 431.66, *p* < 0.001), and the interaction of sex and time after weaning (F_(1,15)_ = 4.53, *p* = 0.05), with females tending to have lower cortisol concentrations than males 4 h after weaning (103.7 ± 4.5 ng/mL *vs.* 116.0 ± 4.4 ng/mL, *p* = 0.07) and both sexes showing lower cortisol concentrations at 28 h compared to 4 h after weaning (*p* < 0.001).

**Table 2:** Physiological variables according to time after weaning and treatment (Least-square means ± standard errors, ^ab^: values with different subscripts differed at *p* < 0.05). *N* = 24 per treatment for each time after weaning (4 or 28 h).

Variables	4 h			28 h		
	Intranasal oxytocin	Subcutaneous oxytocin	Control	Intranasal oxytocin	Subcutaneous oxytocin	Control
Cortisol (ng/mL)	111.1 ± 5.3^a^	108.1 ± 5.8^a^	110.4 ± 5.3^a^	22.7 ± 5.3^b^	17.1 ± 5.3^b^	17.3 ± 5.3^b^
Neutrophil:lymphocyte ratio (arbitrary units)	3.10 ± 0.26^a^	3.85 ± 0.25^a^	3.29 ± 0.27^a^	0.95 ± 0.25^b^	0.85 ± 0.24^b^	1.39 ± 0.25^b^
C-reactive protein (mg/mL)	0.97 ± 0.18	1.14 ± 0.19	0.88 ± 0.23	1.01 ± 0.15	0.96 ± 0.15	1.02 ± 0.15
Tumor Necrosis Factor α (pg/mL)^1^	40.1 ± 50.7	22.6 ± 31.6	31.4 ± 47.8	121.4 ± 33.8	78.3 ± 37.8	28.7 ± 34.7

^1^ For this variable, samples from only 62 pigs were analyzed (IN: N = 20; SC: N = 22; Controls: N = 20).

#### 3.2.2. Neutrophil:lymphocyte Ratio

The neutrophil:lymphocyte ratio did not differ according to treatment (F_(2,12)_ = 1.06, *p* = 0.38), or sex (F_(1,6)_ = 0.44, *p* = 0.38), but differed according to the time after weaning (F_(1,15)_ = 128.74, *p* < 0.001), and tended to differ according to the interaction of treatment and time after weaning (F_(2,15)_ = 2.66, *p* < 0.10; Table 2), with all treatments showing a lower neutrophil:lymphocyte ratio at 28 h compared to 4 h after weaning (all *p* < 0.001), but with no differences between treatments at either time (4 h: *p* = 0.13 and 28 h: *p* = 0.28). Cortisol concentration and neutrophil:lymphocyte ratio were positively correlated (r = 0.64, *p* < 0.001).

#### 3.2.3. C-Reactive Protein

Plasma CRP concentration did not differ according to treatment (F_(2,12)_ = 0.17, *p* = 0.85; Table 2), sex (F_(1,6)_ = 0.18, *p* = 0.69), or time after weaning (F_(1,15)_ = 0.00, *p* = 1.00). C-reactive protein did not correlate with cortisol concentration or neutrophil:lymphocyte ratio (r = −0.07, *p* = 0.49 and r = 0.06, *p* = 0.59; respectively).

#### 3.2.4. Tumor Necrosis Factor α

Plasma TNF α concentration did not differ according to treatment (F_(2,12)_ = 0.72, *p* = 0.51; Table 2), sex (F_(1,6)_ = 0.66, *p* = 0.45), or the time after weaning (F_(1,15)_ = 1.87, *p* = 0.19). Tumor Necrosis Factor α did not correlate with cortisol concentration, neutrophil:lymphocyte ratio, or CRP concentration (r = −0.25, *p* = 0.17; r = −0.23, *p* = 0.22; r = 0.20, *p* = 0.28; respectively).

### 3.3. Behavior

#### 3.3.1. Feeding Behavior

The frequency of feeder visits tended to differ according to treatment (F_(2,12)_ = 2.88, *p* < 0.10; Table 3), with SC pigs visiting the feeder more frequently than IN pigs (*p* = 0.08), but with no difference between IN or SC pigs and control pigs. The frequency of feeder visits did not differ according to sex (F_(1,6)_ = 0.02, *p* = 0.89). The mean duration of feeder visits did not differ according to treatment (F_(2,12)_ = 1.99, *p* = 0.18), or sex (F_(1,6)_ = 0.01, *p* = 0.92).

**Table 3:** Feeding and drinking behavior according to treatment (Least square means ± standard errors; ^xy^: values with different subscripts tended to differ at *p* < 0.10). *N* = 48 per treatment.

Variables	Intranasal oxytocin	Subcutaneous oxytocin	Control
Feeding frequency (bouts per 4 h)	16.3 ± 2.2^x^	22.2 ± 2.2^y^	19.8 ± 2.2^xy^
Feeding duration (sec per 4 h)^1^	305.2 ± 65.8	414.2 ± 65.8	379.0 ± 65.8
Drinking frequency (bouts per 4 h)	16.1 ± 3.1	11.5 ± 3.1	15.3 ± 3.1
Drinking duration (sec per 4 h)	180.5 ± 34.9	117.5 ± 34.9	128.9 ± 34.9

^1^ Data were analyzed using the square root transformation but are presented as non-transformed.

#### 3.3.2. Drinking Behavior

The frequency of using the drinker did not differ according to treatment (F_(2,12)_ = 0.78, *p* = 0.48; Table 3), but tended to differ according to sex (F_(1,6)_ = 4.04, *p* = 0.09), with males using the drinker more often than females (18.6 ± 3.1 bouts *vs.* 10.0 ± 3.1 bouts). The mean duration of use of the drinker did not differ according to treatment (F_(2,12)_ = 1.14, *p* = 0.35; Table 3), but tended to differ according to sex (F_(1,6)_ = 3.92, *p* < 0.10), with males using the drinker for longer than females (189.0 ± 33.4 sec *vs.* 96.6 ± 33.4 sec).

#### 3.3.3. Social Behavior

The frequency of social behavior did not differ accordingly to treatment (F_(2,12)_ = 2.23, *p* = 0.15), or sex (F_(1,6)_ = 0.65, *p* = 0.45), but tended to differ according to the interaction of treatment and sex (F_(2,12)_ = 2.83, *p* < 0.10), with SC females tending to initiate more social behavior than control females (51.8 ± 7.2 bouts *vs.* 35.2 ± 7.1 bouts, *p* = 0.09), but IN females were not different from control females (49.8 ± 7.0 bouts, *p* = 0.14).

**Figure 2:**
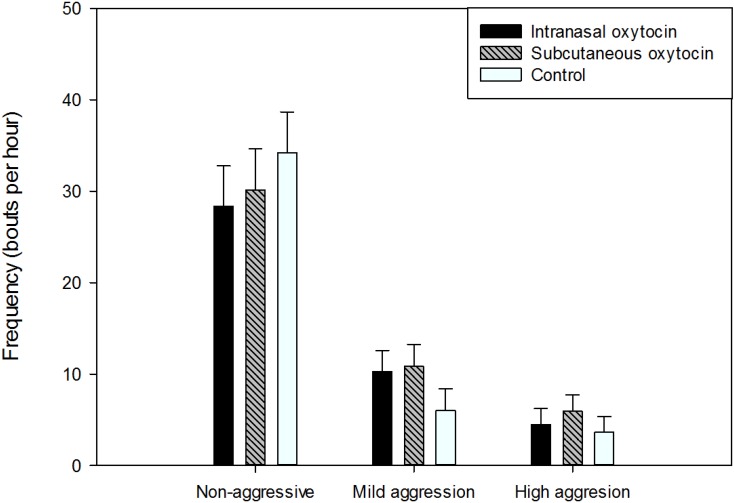
Frequency of the type of social behaviors delivered according to treatment (Least-square means and standard errors). *N* = 24 per treatment.

In terms of type of social behavior, 69.3% were non-aggressive, 20.3% mildly aggressive, and 10.4% highly aggressive social behaviors (Figure 2). The frequency of mild aggression tended to differ according to treatment (F_(2,12)_ = 3.19, *p* = 0.08), with SC pigs tending to deliver more mild aggression than control pigs (*p* = 0.09), but IN pigs were not different from control pigs (*p* = 0.14). The frequency of mild aggression did not differ according to sex (F_(1,6)_ = 1.98, *p* = 0.21). The frequency of high aggression or non-aggressive behaviors did not differ according to treatment, sex, or their interaction.

**Figure 3:**
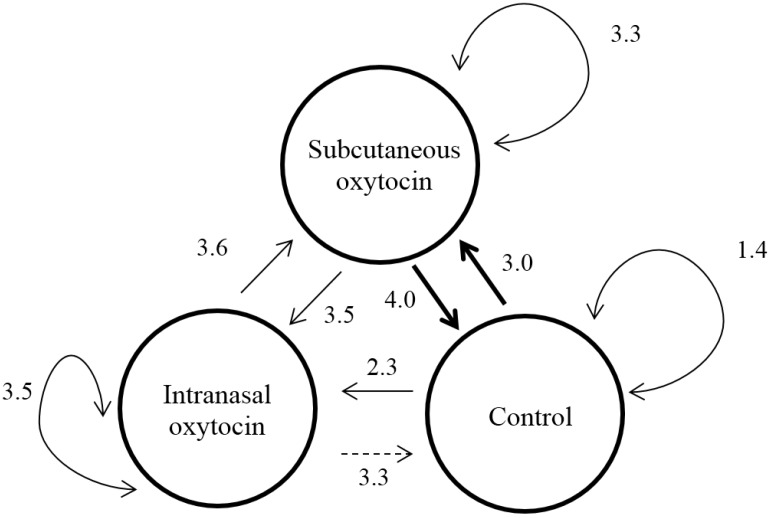
Frequency of mild aggression delivered between treatments (Least-square means, unit is bouts per hour; thick arrows indicate significant differences and dotted arrows trends). *N* = 24 per treatment.

**Figure 4:**
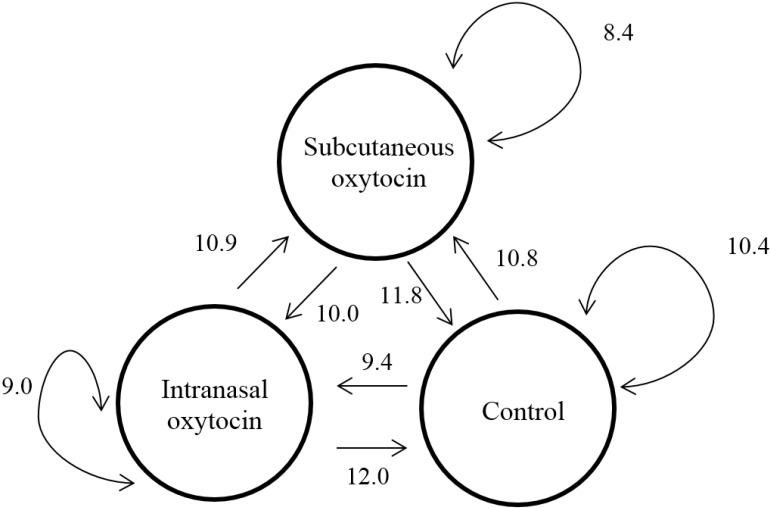
Frequency of non-aggressive behaviors delivered between treatments (Least-square means, unit is bouts per hour). *N* = 24 per treatment.

The target for mild aggression also differed according to treatment (F_(2,12)_ = 4.92, *p* = 0.03; Figure 3), with SC pigs delivering more mild aggression toward control pigs than control pigs to their own (*p* = 0.03) and IN pigs tending to deliver more mild aggression toward control pigs than control pigs to their own (*p* = 0.10). In return, the amount of mild aggression delivered by control pigs also differed according to treatment (F_(2,12)_ = 3.78, *p* = 0.05), with control pigs delivering more mild aggression toward SC pigs than to their own (*p* = 0.04), IN pigs being not different (*p* = 0.28).

Treatments had no effect on the social dynamic of non-aggressive behavior (Figure 4).

## 4. Discussion

To our knowledge, no study has directly compared the effects of acute intranasal and subcutaneous OT administration. Oxytocin administration resulted in higher levels of mild aggression with control pigs, opposite effects on feed intake, and lower body weight 38 days post-weaning for intranasally-administered OT pigs. However, acute OT administration did not result in any noticeable physiological changes 4 or 28 h post-weaning.

Oxytocin administration resulted in higher levels of mild aggression in the group, but did not change non-aggressive, socio-positive behaviors. A previous study showed that intranasally-administered OT pigs were involved in more aggressive encounters and less non-aggressive social contacts [18], although this study could not identify whether OT pigs were the initiator or the recipient of this aggression. The present findings still could not clarify this point: OT-administered pigs engaged in more mild aggressive behaviors, and preferentially toward control pigs rather than OT treated pigs, but OT-administered pigs also received more mild aggression from control pigs. Two possible explanations are that OT administration affects social skills, as evidenced by OT knockout mice [22], resulting in abnormal social behavior; or that OT administration increases aggression. The in-group *vs.* out-group hypothesis has been emitted, in that OT facilitates group cohesion but defensive aggression toward strangers [23]. Unfortunately, we did not record the identity of litters to determine the number and type of social interactions directed toward littermates (two in each pen) *vs.* unacquainted pigs (15 in each pen). The effect of OT administration in established groups *vs.* unacquainted or newly mixed groups is an interesting question, because oxytocin effects are context-dependent [24]. However, OT administration did not affect non aggressive, socio-positive contacts in the present study. Social effects could differ in same treatment groups, *i.e.*, if all penmates were administered OT, as most behavioral changes were observed between rather than within treatments here. A third explanation is that OT may reduce anxiety [25], with weaning triggering an anxious reaction to the novel situation, causing the OT-administered pigs to be less fearful of social confrontation to establish the dominance hierarchy; hence OT switching pigs to a potentially more adaptive social strategy. The subsequent dominance status reached by OT-administered pigs was not tested. It would also be interesting to test whether OT influences unilateral aggression (unilateral biting, head knocks, pursuit), or rather reciprocal aggression (fighting, parallel pushing).

Subcutaneous OT administration resulted in greater effects on social behavior than intranasal OT administration. Both central (intranasal) and peripheral (subcutaneous or intraperitoneal) OT administration have been shown to result in higher central but also peripheral (blood) OT concentrations [26,27]. However, different routes of administration are known to result in different pharmacodynamics, and Neumann and colleagues [27] have shown that peripheral (intraperitoneal) OT administration resulted in quicker changes in central OT concentration and larger changes in plasma OT concentration than intranasal OT administration. In our study, subcutaneous OT was also administered at a dose about three times higher than intranasal OT, which could explain its more potent effects. It cannot be ruled out whether the different sources of OT used for intranasal (95% pure peptide diluted in saline) and subcutaneous administration (Syntocin; OT diluted in preservatives) played a role in determining the effects. The fact that OT administration had more salient effects on the social behavior of females than males is well-known [28].

The reason for opposite effects of OT administration on feed intake according to the mode of administration, with subcutaneous pigs frequenting the feeder more than intranasal pigs, is unclear. Oxytocin administered centrally is known to reduce feed intake [29,30]. Other studies using repeated subcutaneous OT administration have reported no effects or a decrease in feed intake [16,31]. It may reflect different actions according to the triggered physiological pathway. Acute administration of intranasal OT also resulted in slightly lower weight 38 days after weaning. This finding is consistent with the lower feed intake in intranasal OT administered pigs observed after weaning. It suggests a subtle but long-term effect of acute intranasal OT administration. Long-term effects of OT administration have been reported in pigs with repeated postnatal OT administration from one to three days of age dysregulating the hypothalamo-pituitary-adrenal (HPA) axis in the long-term [18]. Nevertheless, that previous study did not find any effect of intranasal OT administration on weight up to slaughter age, much later than the period studied here. Despite the fact that IN pigs showed significantly lighter weights only 38 days after weaning, with the move from the weaner to the grower facility occurring a week prior to that, this weight difference appeared as a progressive change visible from 20 days onwards, rather than an abrupt change triggered by the second move and potential stress.

A single administration of subcutaneous OT did not affect the body weight or growth rate of pigs over the six weeks after weaning. In our previous study [19], subcutaneous OT daily injections to pigs for the first two weeks of life resulted in lower weight loss by two days post-weaning. Despite subcutaneously administered pigs observed more frequently at the feeder over the 4 h after weaning, these pigs did not show any changes in growth rate. Nevertheless, weight loss in the previous study [19] was a lot more severe than in the present experiment, with an average of 250 g/d weight loss whereas the pigs in this present experiment maintained a stable body weight, 3 g/d weight loss in average, over the same period. Hence, stress effects appeared much less pronounced in this study compared to the previous study. This was most likely due to the fact that pigs were housed in groups in the present experiment (common industry situation), but individually after weaning in the previous experiment, which was likely much more stressful and possibly exacerbated the context-dependent actions of OT [24]. It could also be due to the age difference with the pigs weaned at 30 days in the present experiment and 21 days of age in the previous experiment. This lower stress effect may have reduced the chances of detecting an effect of OT administration after weaning in the present experiment. Furthermore, we did not observe any delayed growth advantage as reported for repeated subcutaneously OT administered pigs between three and four weeks post-weaning [19]. Nevertheless, those three studies conducted on the effects of OT administration in pigs differed in the frequency of administration as well as the age of the subjects, which possibly underlies divergent outcomes [32]. The effects of OT on behavior and physiology are complex, and have been shown for instance to differ according to the route of administration [29], doses [33], growth-rate strains in rats [16], or context and individuals [24]. Oxytocin has also been shown to reduce water intake following intracerebroventricular and intraperitoneal administration in rats [30], but no effects could be found here following intranasal or subcutaneous administration.

Weaning in pigs is associated with gut inflammation [34], and OT has been shown to have anti-inflammatory actions. Subcutaneous infusion of OT in rats reduces neutrophils [35], T-lymphocytes [36], and TNF α [35,36], a proinflammatory cytokine with a pivotal role in the inflammation response. However, the single OT administration used in the present study had no effects on leukocytes population or TNF α. Oxytocin was not found to affect plasma CRP, an acute phase protein which is released abundantly as part of a non-specific innate immune system response [37], particular as part of the inflammatory response in pigs [38]. No previous studies could be found that investigated the effects of OT on CRP, although the anti-inflammatory action of OT was expected to reduce CRP. Plasma CRP concentrations were also about 5 to 10 times higher than previously reported serum CRP concentrations [39,40,41], likely due to the fact that CRP was measured in plasma rather than serum as recommended by the kit’s manufacturer.

Oxytocin administration had no effects on stress physiological variables taken 4 and 28 h post-weaning. In contrast to the single dose used here, repeated postnatal OT intranasal administration has been shown to affect the HPA axis in pigs [18]. The finding that females adapt better to weaning than males is consistent with the literature [6,19], and the sharp reduction in cortisol concentration from 4 to 28 h after weaning was expected as pigs adapt to their novel physical and social environment. Cortisol concentration and neutrophil:lymphocyte ratio were positively correlated, supporting that both measures can be used as indicators of the stress response [42,43].

## 5. Conclusions

This study showed little effects of administering OT immediately prior to weaning pigs in order to reduce the stress response observed after weaning. Subcutaneous and intranasal OT administration resulted in higher levels of mild aggression with control pigs, opposite effects on feed intake, and lower body weight 38 days post-weaning for intranasally-administered OT pigs. However, OT administration did not result in any noticeable physiological changes (neutrophil:lymphocyte ratio, plasma cortisol, CRP and TNF-α) 4 or 28 h post-weaning. Hence, these findings do not support the use of a single administration of oxytocin to improve the ability of pigs to cope with weaning, at least not in the conditions studied here.
